# Comparison of Effect of Nebivolol and Bisoprolol on Sexual Function of Hypertensive Female Patients

**DOI:** 10.7759/cureus.15062

**Published:** 2021-05-16

**Authors:** Nomesh Kumar, Shayan Iqbal Khan, FNU Versha, Ishan Garg, Parkash Bachani, Ambresha Gul, Maha Jahangir, Haya Khalid, Sidrah Khan, Sidra Memon

**Affiliations:** 1 Internal Medicine, Liaquat University of Medical and Health Sciences, Jamshoro, PAK; 2 Clinical Medicine, Ross University School of Medicine, Vancouver, CAN; 3 Internal Medicine, Peoples University of Medical and Health Sciences for Women, Nawabshah, PAK; 4 Anesthesiology, Dow University of Health Sciences, Civil Hospital Karachi, Karachi, PAK; 5 Internal Medicine, Jinnah Sindh Medical University, Karachi, PAK; 6 Internal Medicine, Jinnah Postgraduate Medical Center, Karachi, PAK

**Keywords:** nebivolol, bisoprolol, female sexual function, hypertension, sexual function

## Abstract

Introduction

Male and female sexual dysfunction is frequently found in patients with hypertension. Many studies indicate that this is found more frequently in patients treated with beta-blockers rather than due to hypertension itself; however, almost all studies have been done on male population. This study aims to study the effect of two commonly used beta-blockers on sexual function of a hypertensive female patient.

Methods

This two-arm open-label randomized prospective study was conducted from April 1, 2019 to March 30, 2020 in a tertiary care hospital at Pakistan. One hundred and fifty participants randomized to group A were given nebivolol 5 mg once daily in addition to their current hypertensive treatment. Another 150 participants randomized to group B were given bisoprolol 5 mg once daily in addition to their hypertensive therapy. Sexual function was assessed on day 0 and day 90 using female sexual function index (FSFI).

Results

The mean sexual score in the nebivolol group significantly improved after day 90 in comparison to day 0 (24.16 ± 2.1 vs. 26.91 ± 2.6; p-value < 0.0001), while no difference in sexual score in bisoprolol group after day 90 was observed (24.14 ± 2.1 vs. 24.12 ± 2.0; p-value = 0.91).

Conclusion

In this study, nebivolol group was associated with a significant improvement in sexual function. This can be due to additional vasodilation properties and a low risk of sexual side effects associated with nebivolol.

## Introduction

Beta-blocking agents are used for the treatment of various acute and chronic conditions [1]. The primary indication for beta-blockers is cardiovascular pathologies such as hypertension and angina pectoris, but they can also be used to treat anxiety, tremors, migraine, etc. [2]. Other uses of beta-blockers include the treatment of tachycardia, myocardial infarction, cardiac arrhythmias, congestive heart failure, aortic dissection, hyperthyroidism including thyroid storm, anxiety disorders, and glaucoma [3,4].

Beta-blockers exhibit a variety of adverse effects, the most common of which include bronchospasm, heart failure, gastrointestinal symptoms, prolonged hypoglycemia, weight gain, hyperglycemia, bradycardia, heart block, and sexual dysfunction [5,6]. Male and female sexual dysfunction is frequently found in patients with hypertension. Many studies indicate that this is found more frequently in treated patients with hypertension rather than untreated leading to the hypothesis that antihypertensive therapy including beta-blockers might be responsible for this adverse effect [7]. The pathophysiology of male sexual dysfunction due to hypertension and antihypertensive treatment has been largely clarified due to a large number of studies done on the subject, but the pathophysiology of female sexual dysfunction, although being more prevalent than male sexual dysfunction, remains largely uninvestigated, one of the possible reasons being lack of effective treatment [8].

In this study, we will compare the impact of the two most commonly used beta-blockers, i.e., nebivolol and bisoprolol on the sexual function of the hypertensive female patient. This study will be the building block for future studies to investigate the effect of hypertension and its treatment on sexual function in females.

## Materials and methods

This two-arm open-label randomized prospective study was conducted from April 1, 2019 to March 30, 2020 in a tertiary care hospital, Pakistan. Three hundred (n = 300) beta-blocker-naïve hypertensive female patients aged between 20 and 50 years, who were eligible for addition of beta-blockers to their treatment regime, were enrolled from the outpatient department after informed consent. Patients with a history of asthma, hypoglycemia, chronic obstructive pulmonary disease (COPD), electrolyte imbalance, and heart block were excluded from the study, as beta-blockers are contraindicated in these conditions. Patients were randomized by a 1:1 ratio using an online software research randomizer (https://www.randomizer.org/).

One hundred and fifty participants randomized to group A were given nebivolol 5 mg once daily in addition to their current hypertensive treatment. Another 150 participants randomized to group B were given bisoprolol 5 mg once daily in addition to their hypertensive therapy. The patient’s characteristics such as age, gender, history of smoking, duration of hypertension, and family history were noted in the self-structured questionnaire.

A questionnaire was composed using the female sexual function index (FSFI), and questions were inquired in full privacy from the patients. FSFI was translated into the local language, and each question was explained by the interviewer. They were informed that their participation is voluntary and at any moment they can pull out from the study if they are not comfortable. Comprising 19 questions, the FSFI is a validated self-administered questionnaire and evaluates six domains of sexual functions, namely desire, arousal, lubrication, orgasm, satisfaction, and pain. For scoring, the first two questions have scales from one to five and the rest of the questions have scales from zero to five. Scores acquired in a specific domain are added up and multiplied by a respective factor (coefficients for questions 1-2: 0.6; 3-10: 0.3; and 11-19: 0.4), which homogenizes the impact of each dimension. A total of each score is calculated, and healthy sexual life is indicated by a higher score. The scale of the score is between 1.2 and 36. An optimal cut-off value of 26 was introduced. FSFI has high test-retest reliability coefficients, which is high for each of the individual domains (r = 0.79-0.86), and a high degree of internal consistency (Cronbach’s alpha values of 0.82 and higher) [9,10].

Patients were followed up for three months. The questionnaire to assess the FSFI score was repeated on day 90 by the same interviewer. Fourteen participants out of the bisoprolol group and 11 participants of the nebivolol group were lost to follow-up and were excluded from the final analysis.

Statistical analysis was done using Statistical Package for the Social Sciences (SPSS) version 22.0 (IBM Corp., Armonk, New York). Continuous variables were presented as mean and standard deviation. Categorical variables were presented as percentages and frequencies. Chi-square and dependent t-tests were applied to compare categorical data pre- and post-beta-blockers for each group, as appropriate. A p-value of less than 0.05 represented a difference between the case and control group, and the null hypothesis was void.

## Results

In this study, the mean age of participants in the nebivolol group was 42 ± 09 years and 41 ± 09 years in the bisoprolol group. Other hypertensive treatments were comparable between both groups (Table 1).

**Table 1:** Comparison of demographics and treatment ACEI, Angiotensin-converting enzyme inhibitor; ARB, angiotensin receptor blocker; CCB, calcium channel blocker; BMI, body mass index; MI, myocardial infarction.

Characteristics	Patients With Nebivolol (n = 139)	Patients With Bisoprolol (n = 136)	p-value
Age in the p-value year (Mean ± SD)	42 ± 09	41 ± 09	NS
Smoking	15 (11.0%)	12 (8.8%)	NS
Diabetes	41 (29.4%)	37 (27.2%)	NS
BMI greater than 25 kg/m^2 ^	51 (36.6%)	45 (33.0%)	NS
Previous history of acute MI	5 (3.5%)	4 (2.9%)	NS
Family history of acute MI	8 (5.7%)	8 (5.8%)	NS
Other hypertensive treatments			
ACEI	52 (37.4%)	49 (36.0%)	NS
ARBs	71 (51.0%)	68 (50.0%)	NS
CCBs	52 (37.4%)	48 (35.2%)	NS
Diuretics	22 (15.8%)	21 (15.4%)	NS

The mean sexual score in the nebivolol group significantly improved on day 90 (24.16 ± 2.1 vs. 26.91 ± 2.6; p-value < 0.0001). There was no difference in sexual score in the bisoprolol group (24.14 ± 2.1 vs. 24.12 ± 2.0; p-value = 0.91) (Table 2).

**Table 2:** FSFI score of nebivolol and bisoprolol group on day 0 and day 90 FSFI, Female sexual function index.

FSFI Score	Nebivolol Group (n = 139)			Bisoprolol Group (n = 136)		
	Day 0	Day 90	p-value	Day 0	Day 90	p-value
Total sexual score (mean ± SD)	24.16 ± 2.1	26.91 ± 2.6	< 0.0001	25.01 ± 2.2	24.98 ± 2.6	0.91
Participants with score less than 26	71 (51.0%)	42 (30.2%)	0.0006	72 (51.7%)	68 (48.9%)	0.23

## Discussion

Sexuality plays an integral role in the quality of life (QOL). Besides hypertension, antihypertensive medications themselves are a well-known risk factor contributing to female sexual dysfunction (FSD) [11]. Antihypertensive medications like beta-blockers are known to cause sexual dysfunction, hence affecting the QOL, general well-being, and health as a whole. According to the US National Health and Social Life Survey, FSD is more frequent than male sexual dysfunction [12]. Deemed as a complex phenomenon, female sexual function is influenced by numerous biological and psychosocial factors.

Doumas et al. studied the effect of nonselective beta-blockers in female hypertensive patients; they found an increased prevalence of FSD in association with nonselective beta-blockers [12]. Another study suggested that due to the testosterone lowering effect in women, beta-blockers may have negative effects on female sexual function [11]. Studies on the mechanism and effects of beta-blockers as antihypertensive in female sexual function are limited, but literature points toward their similar effects in male and female sexual function. Hence, females should nevertheless receive the same antihypertensive treatment as male patients for FSD [13]. However, there is a relative lack of data regarding the impact of beta-blockers, particularly nebivolol and bisoprolol on sexual function in women.

Blood vessel walls are remodeled due to an increase in blood pressure, leading to impaired vascular supply to the clitoris and vagina, resulting in vasculogenic FSD. Nitric oxide (NO) plays an important role in relaxation of smooth muscles of tunica media, hence improving the blood supply to the genitalia and causing sexual arousal in females similar to erection in males [11,12]. Due to the impairment in NO bioavailability, hypertension results in FSD [12]. Antihypertensive that affects the NO pathway results in improved female sexual function.

Nebivolol, a third-generation agent in addition to its beta adrenoreceptor-blocking activity, has vasodilation properties, arising from its ability to stimulate endothelial release of NO. This mechanism of NO release in response to noradrenergic, non-cholinergic neuronal stimulation leads to relaxation of smooth muscle in the corpus cavernosum, thereby permitting penile erection [14]. Therefore, nebivolol by its mechanism of action may offer an advantage over other beta-blockers when used to treat patients with hypertension. Weiss found no significant difference in hypertensive control between atenolol and nebivolol; however, sexual dysfunction was reported significantly more with atenolol [15]. When compared with placebo, clinical trials showed a similar side effect profile of nebivolol in terms of sexual dysfunction [15].

In patients presenting with sexual dysfunction during treatment with beta-blockers, owing to the low risk of sexual side effects, switching to nebivolol has shown significant improvements in male and female sexual function [13]. This is consistent with Doumas et al. who noted significant improvement in erectile function without a significant change in blood pressure upon switching to nebivolol after six months of therapy with bisoprolol [16]. Despite similar efficacy as antihypertensive, an eight-week treatment regimen with nebivolol resulted in a statistically significant improvement of baseline endothelial function compared with the flow-mediated endothelial-dependent vasodilation values attained after bisoprolol treatment [17]. Hence, we speculate that significant improvement was observed in our study in the mean sexual score in females receiving nebivolol therapy due to their NO-mediating property, and no difference in sexual score in the bisoprolol group was observed.

This is a rare study from this region that studies the role of beta-blockers in FSD. Since the study was conducted in one institute, the sample size was less diverse. So we need further large-scale studies to understand the pathogenesis of sexual dysfunction in hypertensive females and the impact of beta-blockers on them.

## Conclusions

Bisoprolol and nebivolol have similar efficacy for treating hypertension. However, owing to the additional vasodilating properties and low risk of sexual side effects, nebivolol has superiority over bisoprolol and other beta-blocking agents. Hence, it is wise to switch to nebivolol in case of FSD in female patients undergoing antihypertensive treatment with beta-blockers.
